# Multimodal imaging approach to track theranostic nanoparticle accumulation in glioblastoma with magnetic resonance imaging and intravital microscopy†

**DOI:** 10.1039/d5nr00447k

**Published:** 2025-03-19

**Authors:** Giovanni Marco Saladino, Dilyana B. Mangarova, Kerem Nernekli, Jie Wang, Giacomo Annio, Zahra Shokri Varniab, Zubeda Khatoon, Goreti Ribeiro Morais, Yifeng Shi, Edwin Chang, Laura J. Pisani, Grigory Tikhomirov, Robert A. Falconer, Heike E. Daldrup-Link

**Affiliations:** a Department of Radiology, School of Medicine, Stanford University Stanford CA 94305 USA gmsaladino@stanford.edu heiked@stanford.edu; b Institute of Cancer Therapeutics, Faculty of Life Sciences, University of Bradford Bradford BD7 1DP UK; c Department of Electrical Engineering and Computer Sciences, University of California, Berkeley 94720 USA

## Abstract

Theranostic nanoparticles (NPs) have been designed for simultaneous therapeutic and diagnostic purposes, thereby enabling personalized cancer therapy and *in vivo* drug tracking. However, studies thus far have focused on imaging NP tumor accumulation at the macroscopic level and correlating results with *ex vivo* histology. Limited evidence exists on whether *in vivo* NP tumor contrast enhancement on magnetic resonance imaging (MRI) correlates with *in vivo* NP tumor accumulation at the microscopic level. To address this gap, the purpose of our study was to correlate quantitative MRI estimates of NP accumulation with *in vivo* NP signal quantification as measured through two-photon intravital microscopy (IVM) in an orthotopic murine glioblastoma multiforme model (GBM). To enable multimodal imaging, we designed dual-mode NPs, composed of a carbohydrate-coated magnetic core (Ferumoxytol) as an MRI contrast agent, and a conjugated fluorophore (FITC) for IVM detection. We administered these NPs with or without a conjugated vascular disrupting agent (VDA) to assess its effect on NP delivery to GBM. We correlated *in vivo* MRI contrast enhancement in tumors, quantified as *T*_2_ relaxation time, with IVM fluorescence spatial decay rate. Results demonstrated a significantly lower tumor *T*_2_ relaxation time and spatial decay rate in tumors targeted with VDA-conjugated NPs compared to unconjugated NPs. *Postmortem* histological analyses validated the *in vivo* observations. The presented multimodal imaging approach enabled a quantitative correlation between MRI contrast enhancement at the macroscopic level and NP accumulation in the tumor microenvironment. These studies lay the groundwork for the precise evaluation of the tumor targeting of theranostic NPs.

## Abstract 2

Magnetic resonance imaging and two-photon intravital microscopy metrics were correlated to track Ferumoxytol-based theranostic nanoparticles targeting glioblastoma multiforme *in vivo* at both macroscopic and microscopic levels.

## Graphical abstract

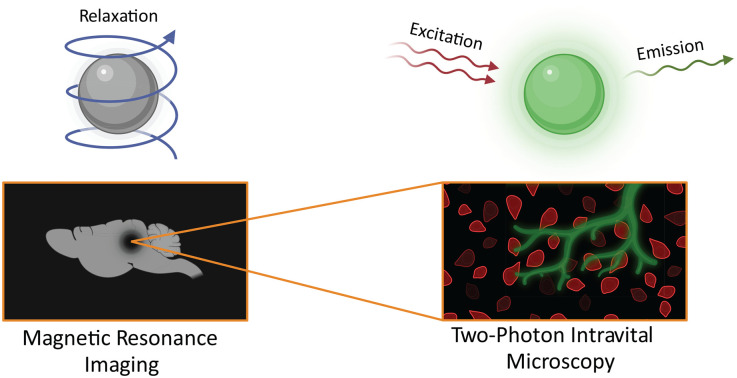

## Introduction

Dual-mode nanoparticles can be employed for multiscale imaging by combining macroscopic and microscopic imaging, thus enabling the study of NP biodistribution at the cellular and whole-tissue levels. Huang *et al.* employed AgAuSe quantum dots embedded in neural stem cell membranes to perform near-infrared imaging for monitoring the multiscale delivery process of the nanoformulation.^1^ Saladino *et al.* designed dual-mode contrast agents for multiscale imaging with whole-body X-ray fluorescence imaging and optical fluorescence histology.^2^ Mannucci *et al.* investigated the biodistribution of fluorescent solid lipid NPs by a multimodal imaging approach correlating *in vivo* whole-body imaging and *ex vivo* microscopy.^3^ Hubert *et al.* employed *in vivo* contrast-enhanced MRI (with Ferumoxytol) and *ex vivo* high-resolution MRI and histological analysis to study neuroinflammatory disorders.^4^ However, none of these studies investigated the microscopic effects of the NPs *in vivo*. In, fact studies to date have focused on imaging NP accumulation in tumors at the macroscopic level and correlating the results with *ex vivo* histology.^5,6^ Limited evidence exists, if *in vivo* NP tumor contrast enhancement on MRI correlates with *in vivo* NP tumor accumulation.^7–9^

Ferumoxytol is a NP formulation (Feraheme™) which is FDA-approved for the treatment of anemia in patients with chronic kidney disease.^10–12^ It is composed of superparamagnetic iron oxide NPs coated with a semi-synthetic carbohydrate.^11^ Ferumoxytol has been proposed as an MRI contrast agent^13,14^ and was demonstrated to provide negative (dark) contrast enhancement of glioblastoma multiforme (GBM) on *T*_2_-weighted MRI scans in patients.^15,16^ Gill *et al.* demonstrated the activity of an matrix metalloproteinase 14- (MMP14-) cleavable vascular disrupting agent (VDA), azademethylcolchicine, against several tumor types supporting its activation in clinical tumors associated with MMP14 expression.^17,18^ However, the direct effects on tumor vasculature were not shown.

Ansari *et al.* reported that Ferumoxytol conjugated to azademethylcolchicine *via* an MMP14-cleavable linker (Ferumoxytol-FITC-VDA) induced significant necrosis in MMP-14 expressing glioblastomas and not normal brain.^19^ Furthermore, Mohanty *et al.* demonstrated that Ferumoxytol-FITC-VDA significantly impaired tumor growth and improved survival of experimental mice.^20^ None of these studies correlated NP-mediated tumor contrast enhancement on MRI with *in vivo* NP tumor accumulation at the microscopic level.

Two-photon intravital microscopy (IVM) is an optical imaging technique designed to study living organisms *in vivo* at the microscopic level, which can detect specific cells and drug carriers relying on fluorescent molecular probes that are bound to the target.^21^ Recently, IVM has been used to investigate NP interactions within the biological environment,^22,23^ Naumenko *et al.* studied biodistribution of PEGylated magnetic NPs in kidney with IVM.^24^ Dong *et al.* employed IVM to study the delivery of therapeutic NPs to the brain in real-time.^25^ Pellow *et al.* performed simultaneous intravital optical and acoustic monitoring for studying nanobubble extravasation.^26^ Zhang *et al.* investigated the use of FITC-labeled silicon NPs in a syngeneic orthotopic glioma mouse model to assess their tumor-targeting abilities with IVM.^27^ None of these studies correlate the findings with *in vivo* contrast MRI. Therefore, the purpose of our study was to correlate quantitative MRI estimates of theranostic NP accumulation in glioblastoma multiforme (GBM) with *in vivo* NP signal on two-photon intravital microscopy (IVM) in an orthotopic murine GBM model.

## Results

### Comparison of physicochemical characteristics

**Fig. 1:**
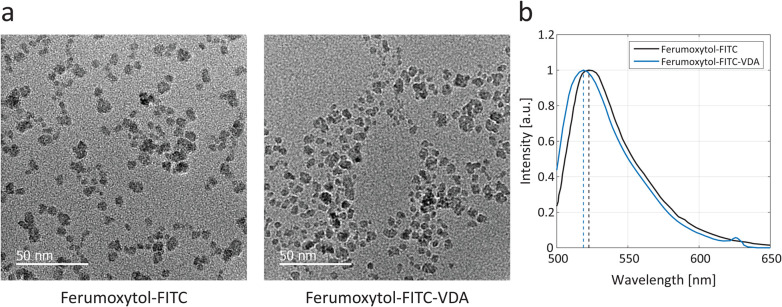
Electron microscopy and optical fluorescence. (a) TEM micrograph of Ferumoxytol-FITC showing the dispersed iron oxide cores. Scale bars, 50 nm. (b) Optical fluorescence spectra of Ferumoxytol-FITC and Ferumoxytol-FITC-VDA, employing an excitation wavelength of 480 nm, and yielding emission peaks at 521 and 517 nm, respectively.

Ferumoxytol-FITC and Ferumoxytol-FITC-VDA were characterized with transmission electron microscopy (TEM, Fig. 1a). Both NPs exhibited the characteristic morphology of Ferumoxytol (Fig. S1a†), showing dispersed NPs with diameters ranging from 5 to 10 nm. Ferumoxytol-FITC and Ferumoxytol-FITC-VDA yielded emission peaks at 521 and 517 nm, respectively, under an excitation wavelength of 480 nm (Fig. 1c). Dynamic light scattering (DLS) and polydispersity index (PDI) demonstrated the variation of the hydrodynamic size following surface functionalization of Ferumoxytol. The unconjugated Ferumoxytol exhibited a size of 25.4 nm (PDI 0.102). Upon conjugation with the sole fluorophore (FITC, Fig. S1b†), the hydrodynamic size of the conjugated Ferumoxytol-FITC coherently increased to 30.6 nm (PDI 0.106). The hydrodynamic diameter of Ferumoxytol-FITC-VDA (Fig. S1c†) was estimated as 34.4 nm (PDI 0.151), consistent with the conjugation of a larger molecule (ICT3105) than FITC.

The FT-IR spectra of the conjugated NPs were compared to the bare Ferumoxytol and free molecules (Fig. S2, *cf.* ESI†).^28–31^ The effect of the synthesized NPs on C6 glioblastoma cells was also tested with a cell-counting kit (CCK-8) viability assay, an indicator of metabolic activity, before proceeding to *in vivo* evaluations (Fig. S3, *cf.* ESI†).^17,32,33^

**Fig. 2:**
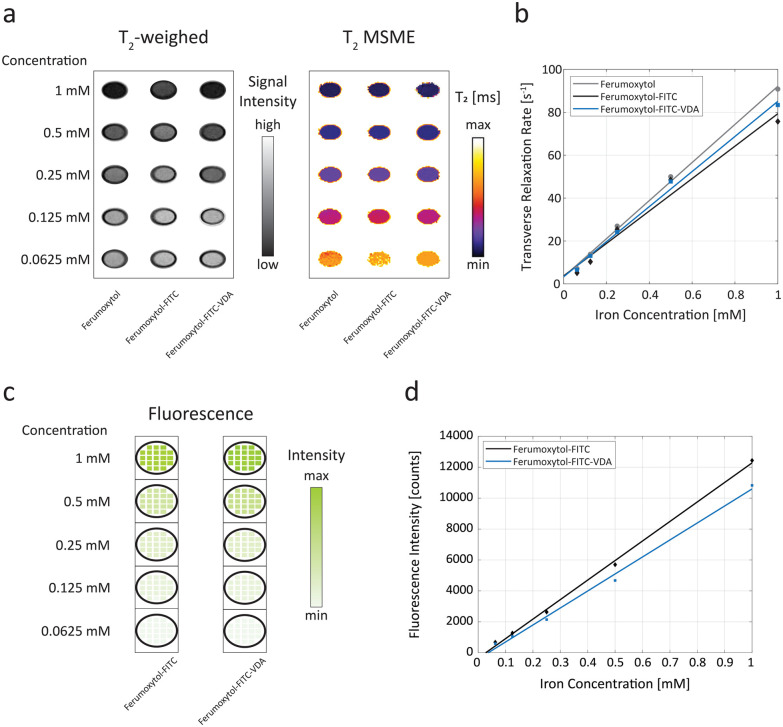
Dual-mode nanoparticle properties. (a) Representative axial *T*_2_-weighted scan of phantoms with increasing concentrations of nanoparticles and corresponding *T*_2_ multi-slice multi-echo (MSME) map. (b) Transverse relaxation rate (*R*_2_) as a function of iron concentration for Ferumoxytol (in grey), Ferumoxytol-FITC (in black), and Ferumoxytol-FITC-VDA (in blue). (c) Optical fluorescence map demonstrating increasing fluorescence of FITC-conjugated nanoparticles with increasing iron concentration. (d) Fluorescence intensity as a function of iron concentration for Ferumoxytol-FITC (in black), and Ferumoxytol-FITC-VDA (in blue).

To assess the dual-mode properties of the designed NPs, we conducted a series of phantom tests using both MRI and optical fluorescence techniques. By varying the concentrations of the NPs in dispersion, their performance was evaluated (Fig. 2).

The transverse relaxivities (*r*_2_) of Ferumoxytol-FITC and Ferumoxytol-FITC-VDA were 65 ± 28 s^−1^ mM^−1^ and 77 ± 14 s^−1^ mM^−1^, respectively. Their difference was not statistically significant at the 5% significance level. The transverse relaxivity of the bare Ferumoxytol was 88 ± 14 s^−1^ mM^−1^ (Fig. 2a and b). The estimated values of the longitudinal (*r*_1_) relaxivities were 1.9 ± 0.4 s^−1^ mM^−1^ and 1.6 ± 0.3 s^−1^ mM^−1^ for Ferumoxytol-FITC and Ferumoxytol-FITC-VDA, respectively. The *r*_1_ relaxivity of Ferumoxytol alone was 2.6 ± 0.7 s^−1^ mM^−1^ (Fig. S4a and b†). Ferumoxytol-FITC and Ferumoxytol-FITC-VDA exhibited a linearly increasing fluorescence intensity with increasing iron concentration, with Pearson correlation coefficients of 0.990 and 0.997, respectively (Fig. 2c and d).

### Magnetic resonance imaging

**Fig. 3:**
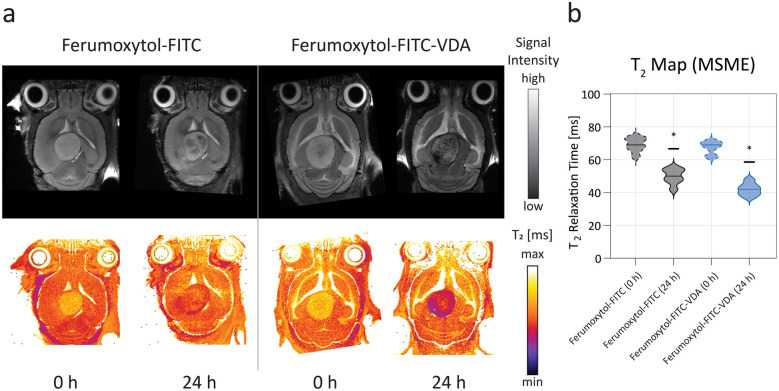
*In vivo* magnetic resonance imaging before and after NP injection. (a) Representative axial *T*_2_-weighted MR images of the brain and corresponding color *T*_2_ maps of mice before (0 h) and after (24 h) after intravenous injection of either Ferumoxytol-FITC (50 mgFe per kg) or Ferumoxytol-FITC-VDA (50 mgFe per kg). (b) Mean *T*_2_ relaxation time violin plots of the tumor area from mice injected with either Ferumoxytol-FITC (in black) or Ferumoxytol-FITC-VDA (in blue), before (*t* = 0 h, dashed lines) and after (*t* = 24 h, solid lines) NP administration. Significant difference between the two groups was indicated when *P < 0.05 (*n* = 4 per group).

Pre-contrast MRI did not show a significant difference of the tumor *T*_2_ signal (*P* > 0.05) between control (pre-Ferumoxytol-FITC) and treatment (pre-Ferumoxytol-FITC-VDA) groups (*t* = 0 h), as expected (Fig. 3a), with a mean tumor *T*_2_ relaxation time of 69 ± 4 ms and 67 ± 4 ms, respectively (Fig. 3b). At 24 hours after intravenous administration of Ferumoxytol-FITC and Ferumoxytol-FITC-VDA, all brain tumors demonstrated hypointense (dark) contrast enhancement on the *T*_2_-weighted images, indicating NP accumulation in the tumor. Mice administered with Ferumoxytol-FITC-VDA demonstrated a significant (*P* < 0.05) negative contrast enhancement in the tumor (42 ± 4 ms) compared to tumors of mice injected with Ferumoxytol-FITC (50 ± 5 ms).

### Intravital microscopy

**Fig. 4:**
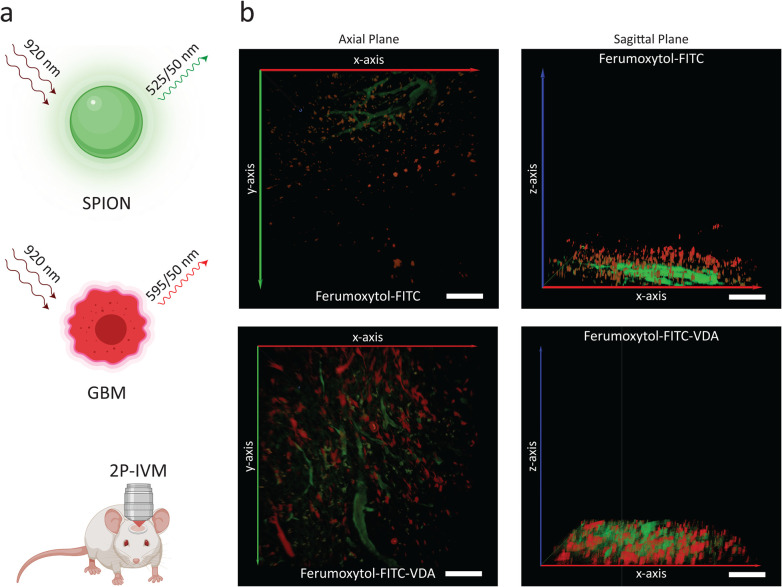
*In vivo* two-photon intravital microscopy of GBM after NP injection. (a) Schematic representation of the excitation laser and detection windows for conjugated iron oxide nanoparticles (SPIONs), in green) and implanted glioblastoma multiforme (GBM, in red), for imaging through two-photon intravital microscopy (IVM). (b) Representative IVM images of implanted brain tumors in mice injected with Ferumoxytol-FITC and Ferumoxytol-FITC-VDA at 24 h after NP administration, showing GBM (in red) and FITC (in green) signals in axial planes and corresponding sagittal planes. Scale bars, 100 μm.

IVM enabled *in vivo* imaging of the tumor microenvironment after intravenous injection of Ferumoxytol-FITC or Ferumoxytol-FITC-VDA (Fig. 4). With the laser mode-locked at 920 nm, NPs were imaged through two-photon excitation of FITC, leading to green emission (Fig. 1b). Red fluorescent protein- (RFP-) labeled C6 tumor cells could be detected with a band-pass filter (595/50 nm) in the red spectrum (Fig. 4a).

The vasculature architecture was indirectly observed from the FITC signal of the NPs. The control group (Ferumoxytol-FITC) highlighted typical vasculature (Fig. 4b). Tumors treated with Ferumoxytol-FITC-VDA led to a pronounced breakdown of the vascular architecture, observed as structural irregularities or branching tumor vessels and leak of green fluorescent NP signal into the tumor interstitium. Furthermore, with *z*-stacking, it was possible to observe a more diffused signal of the theranostic NPs compared to the control. In this respect, NP extravasation was quantitatively investigated by analyzing the exponential decay rate of the fluorescent signal from intensity profiles across the blood vessels (Fig. S5a and b†).

**Fig. 5:**
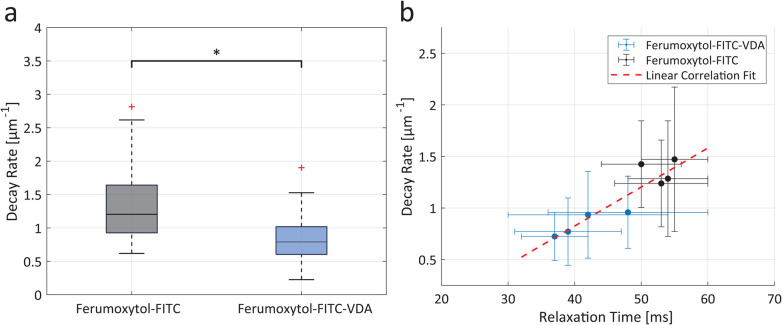
Nanoparticle extravasation and multiscale imaging correlation. (a) Boxplot of fluorescence spatial decay rate of Ferumoxytol-FITC (in grey) and Ferumoxytol-FITC-VDA (in blue). Significant difference between the two groups was indicated when *P < 0.05 (*n* = 4 per group). (b) Correlation scatter plot between spatial decay rate and *T*_2_ relaxation time for mice administered with Ferumoxytol-FITC (in black) and Ferumoxytol-FITC-VDA (in blue). Experimental points (±SD) were fitted with a linear regression (dashed red line, *R*^2^ = 0.84).

The fluorescence spatial decay rate of Ferumoxytol-FITC-VDA (0.8 ± 0.4 μm^−1^) was significantly lower than Ferumoxytol-FITC (1.3 ± 0.6 μm^−1^), indicating that Ferumoxytol-FITC-VDA diffused further away from the blood vessels (Fig. 5a, *P* < 0.05). The tumor *T*_2_-relaxation time (MRI) correlated significantly with the spatial decay rate (IVM) with a positive association between the two variables and a Pearson correlation coefficient of 0.915 (Fig. 5b).

### Histopathological validation

**Fig. 6:**
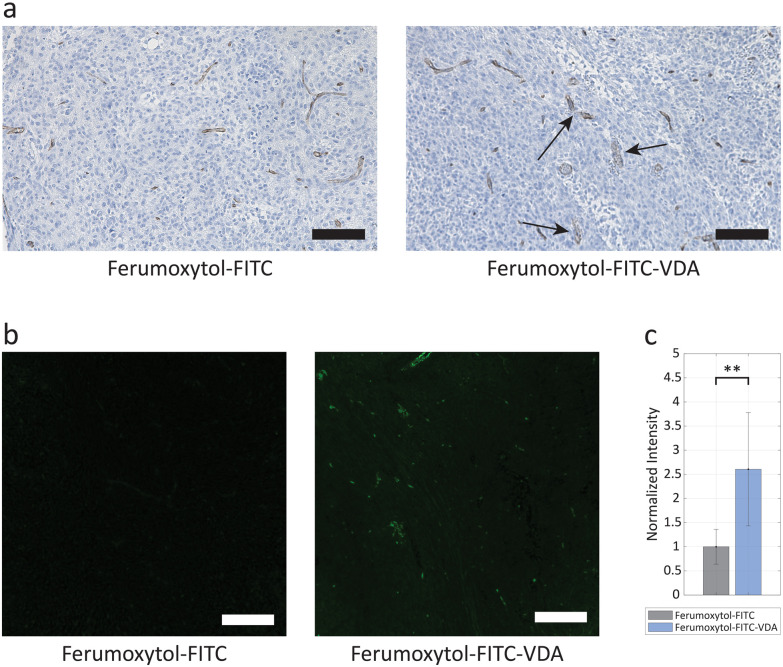
Histopathology analysis. (a) CD31 staining (in brown) of mouse brain tumor tissue following injection with Ferumoxytol-FITC or Ferumoxytol-FITC-VDA, highlighting signs of vessel collapse (black arrows). Scale bars, 200 μm. (b) Optical fluorescence analysis of FITC signal from tumor tissue slides of mice injected with Ferumoxytol-FITC or Ferumoxytol-FITC-VDA. Scale bars, 200 μm. (c) Normalized intensity (±SD) bar plot comparing Ferumoxytol-FITC and Ferumoxytol-FITC-VDA. Significant difference between the two groups was indicated when **P < 0.005.

CD31, a marker for endothelial cells was used to identify the blood vessels in the tumor tissue at 24 h after intravenous injection of Ferumoxytol-FITC or Ferumoxytol-FITC-VDA. Tumor sections stained with CD31 demonstrated typical vasculature in the former and altered vasculature with fragmented CD31-positive staining and brown particulate in the latter (Fig. 6a). Fluorescence microscopy images of unstained tumor tissue sections demonstrated the presence of diffused FITC signal after administration of Ferumoxytol-FITC and Ferumoxytol-FITC-VDA (Fig. 6b). The green fluorescence signal was, on average, 2.6 times higher (*P* < 0.005) in tumors from mice injected with Ferumoxytol-FITC-VDA than in those from mice administered Ferumoxytol-FITC (Fig. 6c).

MMP14-immunostains revealed low MMP14 expression of the healthy brain and strong MMP14 expression of GBM (Fig. S6a†), with fluorescence intensity 6.5 times higher than healthy tissue, on average (Fig. S6b†).

## Discussion

Data showed a strong correlation of quantitative MRI estimates (*T*_2_ relaxation time) of theranostic NP accumulation in GBM with *in vivo* NP signal on two-photon IVM. The correlation could take advantage of the specific characteristics of two complementary techniques: MRI facilitates the detection of NP accumulations and anatomic soft tissue features, by intensity variations, enabling an easy macroscopic localization within the tissue;^34^ Ferumoxytol was selected as the MRI *T*_2_ contrast agent due to its status as an FDA-approved NP formulation for the treatment of iron deficiency in patients with anemia.^35^ We used Ferumoxytol off label as an MR contrast agent in pediatric patients and found very limited adverse effects in pediatric patients.^36^ IVM can provide advanced studies of the *in vivo* biodistribution of NPs with multiple channels and multiplexed images, differentiating between several fluorescent targets at a microscopic level, here exemplified by the tumor cells and NPs. Denk *et al.* highlighted the advantages of two-photon microscopy, including the minimization of tissue autofluorescence, compared to single-photon microscopy, owing to the longer excitation wavelengths (in the near-infrared spectrum) and localized excitation (at the focal point).^37^ For this reason, we hereby chose two-photon excitation with IVM.

The transverse relaxivity (*r*_2_) was not significantly affected by the conjugation of the Ferumoxytol surface with either ICT3105 or FITC, when compared to bare Ferumoxytol. In fact, Zhang *et al.* demonstrated that the transverse relaxivity depends largely on the magnetization of the contrast agent, rather than its surface.^38^ On the other hand, the longitudinal relaxivity (*r*_1_) was more strongly affected by the conjugation of FITC and ICT3105. It is known that accessibility of water to the paramagnetic center of the NPs is crucial for *T*_1_ contrast enhancement,^38^ which was partially hindered by the conjugated molecules. We utilized the similar *r*_2_ relativities of Ferumoxytol-FITC and Ferumoxytol-FITC-VDA for comparison between the treatment and the control groups, with *in vivo* MRI studies.

The optical fluorescence properties were provided by the conjugated FITC, leading to fluorescent NPs with emission peaks in agreement with FITC characteristic peak.^39^ Ferumoxytol-FITC and Ferumoxytol-FITC-VDA had slightly larger hydrodynamic diameter than Ferumoxytol, which was ascribed to the FITC conjugation, and the unaltered PDI indicates that NPs did not undergo agglomeration upon the conjugation and washing steps. Together, the magnetic and fluorescence characterizations demonstrated the potential of the conjugated NPs for multiscale dual-mode imaging with MRI and IVM.

Ferumoxytol biodistribution and biosafety have been extensively studied and established in preclinical and clinical trials prior to its approval by the FDA for the treatment of iron deficiency.^40^ Furthermore, clinical studies have investigated Ferumoxytol metabolism and clearance in the liver of healthy adults.^41^ T2-signal of the liver on MR images was back to baseline between 3–11 months after NP administration. Mohanty *et al.* studied the Ferumoxytol-FITC-VDA distribution through longitudinal studies over the course of 2 weeks.^20^ In the present work, we investigated Ferumoxytol delivery to GBM orthotopic tumors.

The accumulation of Ferumoxytol-FITC in brain tumors was ascribed to the enhanced permeability and retention (EPR) effect (passive targeting),^42,43^ yielding only limited NP accumulations within the tumor area, mainly affecting the tumor margins, as visualized with the MRI axial brain slices. Ansari *et al.* used a combination of *in vivo* MRI and *ex vivo* histology, and reported that Ferumoxytol-FITC-VDA induced tumor-selective accumulations and tumor necrosis, thus qualifying as a theranostic agent.^19^ Ansari *et al.* also demonstrated that the MRI tumor contrast enhancement 24 hours after NP administration was higher than that observed 1 hour post-administration.^19^ Therefore, we selected the 24-hour time point for our correlative IVM-MRI studies. Future longitudinal studies could provide further insights into the biodistribution of theranostic NPs and enable the assessment of the IVM-MRI correlation at different timepoints.

MRI alone could not provide mechanistic evidence of the microscopic NP distribution within the brain vasculature and tumor microenvironment. Tissue histology includes fixation, embedding, and sectioning potentially introducing artifacts and, thus, it may not reflect the tissue's true state. The presented *in vivo* multimodal imaging approach could correlate results between macroscopic observations and changes in the tumor microenvironment, where IVM enabled the observation of tissues in the native state.

Mohanty *et al.* demonstrated that Ferumoxytol-FITC-VDA significantly impaired growth of GBM and induced apoptosis of GBM-initiating cells. Furthermore, the combination therapy with Ferumoxytol-FITC-VDA and temozolomide achieved tumor remission and increased survival of mice by more than two folds, compared to treatment with only temozolomide.^20^ In the present work, IVM could track NP accumulation in tumor as diffused FITC signal in the perivascular regions, indicating NP diffusion through the disrupted endothelial barriers provoked by the VDA. The compromised vascular integrity, hence, facilitated the NP leakage into the surrounding tissue. This observation provided direct evidence of the successful release and action of the active VDA (ICT3105), which was delivered to the tumor and activated by MMP14.

In a recent study, Wang *et al.* demonstrated that multiphoton microscopy could distinguish the degree of extravasation for simultaneously administered NPs with varying core sizes, by analyzing the fluorescence intensity curves.^44^ In our study, NP extravasation was quantitatively investigated by analyzing the spatial decay rate of the fluorescent signal with IVM, permitting to investigate one NP type at a time. Schubert *et al.* employed intravital microscopy to study glioblastoma progression *in vivo*,^45^ but did not investigate the action of contrast agents or therapies. Our results substantiated the macroscopic observations made with whole-tumor MRI and provided microscopic mechanistic evidence of the theranostic NP interactions with the tumor microenvironment. The low mean decay rates (<1 μm^−1^) and relaxation times (<50 ms) achieved by the administration of theranostic NPs provided evidence of the improved targeting efficacy of Ferumoxytol-FITC-VDA, compared to Ferumoxytol-FITC. Furthermore, the correlation between MRI and IVM metrics was demonstrated with both passive (Ferumoxytol-FITC) and active targeting (Ferumoxytol-FITC-VDA) NPs. Our correlative imaging approach could be used to investigate the tumor delivery and retention of other commercially available and theranostic NPs, such as MegaPro NPs,^46^ Ocean NanoTech NPs,^47^ and clinically-translatable theranostic NPs.^48^

To validate the observations made with IVM within the tumor microenvironment, the extracted brain tissue was processed for histopathological analysis with the CD31 marker for endothelial cells, which was employed for the identification of the blood vessels. Tumor vasculature, following the action of the MMP14-cleavable VDA, showed fragmented and irregular CD31-positive staining together with brown particulate (endothelial cell debris), indicating significant endothelial cell damage and vessel collapse.^49^ These observations validated the findings made with IVM, confirming the impact of VDA on tumor blood vessels and theranostic properties of Ferumoxytol-FITC-VDA. The green fluorescence signal from unstained tissue slides of tumor slices confirmed that the increased NP extravasation detected with IVM and lower relaxation times in MRI reflect a larger NP uptake by the tumor. In future experiments, Evans blue (EB) or horseradish peroxidase (HRP) leakage assays could quantify the extent of the blood–brain barrier (BBB) permeability.

Immunohistochemistry results for MMP14-staining proved a strong upregulation of MMP14 in the tumor microenvironment,^50^ and causally validated the targeted action of the theranostic NPs, Ferumoxytol-FITC-VDA, towards GBM, with its MMP14-cleavable VDA. Together, the presented *postmortem* analyses with the CD31 marker and MMP14 staining confirmed the observations made through IVM, thus emphasizing the robustness of the presented *in vivo* multimodal correlative imaging approach. In the future, this approach could facilitate the study of the relationship between tumor heterogeneity and the targeting efficacy of theranostic NPs.

## Conclusions

We found a strong correlation of quantitative MRI estimates of theranostic NP accumulation in a murine model of GBM with *in vivo* NP signal on IVM. MRI and IVM techniques together allowed one to visualize the fate of NPs *in vivo* at different scales, attributing a crucial role to dual-mode imaging of nanomedicines, which hereby provided evidence of the successful delivery and activation of the prodrug (VDA). In the future, the presented correlative imaging approach with MRI and IVM could be employed to investigate other theranostic platforms and commercial formulations. Understanding the mechanisms underlying enhanced delivery can provide valuable insights for selecting the most effective diagnostic and therapeutic strategies using theranostic NPs thus facilitating clinical translation.

## Methods

### Materials

Ferumoxytol (Feraheme®) was purchased from AMAG Pharmaceuticals. Sodium hydroxide (NaOH), succinimidyl-([*N*-maleimidopropionamido]-4-ethyleneglycol)ester [SM(PEG)_4_], sodium carbonate, sodium bicarbonate, ammonium hydroxide (NH_4_OH), epichlorohydrin, dimethyl sulfoxide (DMSO), phosphate-buffered saline (PBS), and fluorescein isothiocyanate (FITC) were purchased from Fisher Scientific. Diethyl ether, Fmoc-*O-tert*-butyl-l-tyrosine (Fmoc-Tyr(O*t*Bu)-OH), and triisopropyl silane (TIS) were purchased from Sigma Aldrich. Colchicine and 1,2-ethanedithiol were obtained from Alfa Aesar and Fluka Analytical, respectively. 2-Chlorotrityl chloride and trifluoroacetic acid (TFA) were purchased from Novabiochem and Fluorochem, respectively.

### Nanoparticle functionalization

The Ferumoxytol surface was aminated with epichlorohydrin, as previously described,^51^ with slight modifications: briefly, a mixture of Ferumoxytol (3.5 mgFe per mL), NaOH (2.4 M), and epichlorohydrin (2.5 M) was prepared in distilled water (total volume, 20.5 mL). The dispersion was shaken for 24 h and subsequently filtrated with dialysis (12–14 kDa cutoff) against water for 3 days. The dispersion was then mixed with NH_4_OH (10 mL) and kept stirring at 37 °C for 24 h. Finally, the dispersion was dialyzed against water for 3 days, leading to amine-functionalized Ferumoxytol (Ferumoxytol-NH_2_).

Ferumoxytol nanoparticles were conjugated to FITC by adding a FITC solution (1 mg mL^−1^, DMSO) to Ferumoxytol-NH_2_ in a carbonate–bicarbonate (Na_2_CO_3_/NaHCO_3_) buffer (pH 9) and incubating it at 4 °C for 8 h. The reaction was quenched by adding NH_4_OH and further incubated for 2 h. Finally, the sample was dialyzed against saline (2 days) to remove excess FITC and NH_4_OH.

The vascular disrupting agent ICT3105 was synthesized employing a combination of solution and solid phase peptide synthesis methodologies, as previously reported:^17,18^ in brief, azademethylcolchicine, synthesized from amination of colchicine, was conjugated to commercially-available Fmoc-Tyr(O*t*Bu)-OH, followed by selective acidic hydrolysis of the side-chain *tert*-butyl group and loading onto 2-chlorotrityl-chloride resin. The resin was then used to prepare ICT3105 by Fmoc solid phase peptide synthesis, followed by simultaneous hydrolysis of side-chain protective groups and resin cleavage using a mixture of TFA : TIS : water : 1,2-ethanedithiol (94 : 1 : 2.5 : 2.5, 4 mL per 100 mg resin) at room temperature for 4 h. The solvent was subsequently evaporated, and ICT3105 (Fig. S7†) was precipitated from diethyl ether.

To synthesize Ferumoxytol-FITC-VDA, Ferumoxytol-NH_2_ was reacted with SM(PEG)_4_ in PBS (pH 7.4), concentrated with Microcon® centrifuge filters (10 kDa cutoff), and subsequently incubated with de-protected ICT3105 (7 mg mL^−1^). The final stock was obtained by several filtration steps to remove unreacted molecules, leading to an average of 4.7 conjugated ICT3105 molecules per NP.^19^

### Nanoparticle physicochemical characterization

The concentration of iron (Fe) in Ferumoxytol-FITC and Ferumoxytol-FITC-VDA solutions was determined using an inductively coupled plasma-mass spectrometer iCAP 6300 (Thermofisher Scientific, USA). The hydrodynamic sizes of Ferumoxytol, Ferumoxytol-FITC, and Ferumoxytol-FITC-VDA were measured with dynamic light scattering (DLS) using a Nanobrook Omni (Brookhaven Instrument, USA). The infrared spectra were collected with a Nicolet iS50 FT/IR Spectrometer (Thermo Fisher Scientific, USA). The optical fluorescence spectra were acquired with a FluoroLog Fluorimeter (Horiba, USA). Transmission electron micrographs were acquired with a Tecnai G2 F20 X-TWIN (FEI, USA) at 200 kV. Ferumoxytol, Ferumoxytol-FITC and Ferumoxytol-FITC-VDA were dispersed in Eppendorf tubes with increasing concentrations (0.0625 mM–1 mM); these were scanned on a 7T MRI scanner (Bruker Biospin, Billerica, MA) with *t*_1_-w rapid acquisition with relaxation enhancement (RARE) sequence (TR = 1500 ms, TE = 10 ms, FOV = 60 mm × 60, 256 × 256 acquisition matrix), *t*_2_ RARE sequence (TR = 2200 ms, TE = 21 ms, FOV = 60 mm × 60, 256 × 256 acquisition matrix), *t*_1_ map variable TR (VTR) saturation recovery sequence (TE = 10 ms, TR = 300, 500, 700, 1000, 1500, 2000, 2500, 4000, and 5500 ms, RARE factor = 2, FOV 60 mm × 60 mm, 256 × 256 acquisition matrix, 2 averages), and a multi-slice-multi-echo (MSME) sequence for *t*_2_ map (TR = 2200 ms, TE = 7, 14, 21, 28, 35, 42, 49, 56, 63, 70, 77, 84, 91 and 98 ms, FOV 60 mm × 60 mm, 256 × 256 acquisition matrix, 3 averages). *T*_1_- and *T*_2_ maps were generated with ImageJ software. Transverse and longitudinal relaxation rates as a function of NP concentration were plotted and the relaxivities (*r*_1_ and *r*_2_) were estimated from the mono-exponential signal decay using a linear regression analysis. The optical fluorescence properties were studied as a function of iron concentration with the Synergy H1 hybrid reader (BioTek, USA), using diluted dispersions of Ferumoxytol-FITC and Ferumoxytol-FITC-VDA (0.0625 mM–1 mM), in a 24-well plate. The excitation and emission windows were set to 490 nm and 520 nm, respectively.

### Cytotoxicity studies

Cell viability was assessed using the CCK-8 assay (Dojindo Laboratories, Japan), following the manufacturer's protocol. Briefly, C6 tumor cells were seeded in 96-well plates (10^4^ cells per well) and incubated under standard conditions (37 °C, 5% CO_2_). The cells were treated with either of the three NP types, Ferumoxytol, Ferumoxytol-FITC, and Ferumoxytol-FITC-VDA, at different concentrations (1000, 500, 200, 100, 50, 20, 10, 0 μg mL^−1^). After 24 h incubation, the CCK-8 reagent was added, and the absorbance was measured at 450 nm using a microplate reader. All experiments were done in triplicates. Cell viability was expressed as a percentage relative to (negative) control, calculated by normalizing absorbance values from treated wells to those of untreated control wells.

### Tumor implantation

The animal experiments were approved by the Administrative Panel on Laboratory Animal Care (APLAC) and conducted according to protocol #24965. 5-Month-old female NSG mice (NOD.Cg-Prkdcscid Il2rgtm1Wjl/SzJ, *n* = 8) were housed at room temperature (20–22 °C) with 30–70% humidity, and a 12 h light–dark cycle, with free access to food and water. For tumor cell transplantation into the brain, the mice were anesthetized with isoflurane (3–5% induction, 1–2.5% maintenance) and positioned in a stereotactic frame for precise placement, and the surgery was conducted under sterile conditions. To avoid any intra- and post-operative pain or infection, carprofen (5–20 mg kg^−1^), sustained-release buprenorphine (1 mg kg^−1^), cefazolin (20 mg kg^−1^), and dexamethasone (0.2 mg kg^−1^) were administered subcutaneously before the surgery. A 3 mm section of the right parietal scalp and bone was removed, and 10^6^ tumor RFP-labeled C6 tumor cells were injected at a depth of 1 mm below the brain surface, with intrathecal injection coordinates medial to lateral (M–L): −1.2 to −1.6 mm, and dorsal to ventral (D–V): −2.6 to −3.5 mm. The brain was covered with a glass plate and secured to the skull using an MRI-compatible frame and cyanoacrylate glue.^52^ For recovery, animals were provided with DietGel (ClearH20 Inc., ME, USA) to ensure hydration and nutrition.

### MR imaging

All mice underwent MRI on a 7T MR scanner (Bruker Biospin, Billerica, USA) before and at 24 hours after intravenous injection of either Ferumoxytol-FITC (50 mgFe per kg, *n* = 4) and Ferumoxytol-FITC-VDA (50 mgFe per kg, *n* = 4). Mice were anesthetized with isoflurane (3–5% induction, 1–2.5% maintenance) and placed in a prone position. The anesthetized mice received venous access *via* the tail vein for NP administration. During the imaging session, the body temperature (37 °C) and respiratory rate were constantly monitored. Brain tumors were imaged with *T*_2_-weighted fast spin echo (FSE) using a field of view of 2 cm × 2 cm and a slice thickness of 0.5 mm and the following protocol: repetition time (TR): 4500 ms, echo time (TE): 42 ms, flip angle *α*: 90° and *T*_2_ multi-slice multi-echo (MSME): TR: 3000 ms, TE: 8, 16, 24, 32, 40, 48, 56, 64, 72, 80, 88 and 96 ms, *α*: 90°. *T*_2_ relaxation times were measured to evaluate changes in signal perturbations in the tumor microenvironment post-treatment.

### Intravital imaging

Mice were anesthetized and secured to a custom-built stage to minimize breathing artifact during image acquisition. Images were acquired with a 25× water immersion objective (NA 0.9) on a Leica SP8 2p microscope equipped with a tunable pulsed chameleon infrared multiphoton laser (Coherent, USA) and two high-sensitivity hybrid-PMT (HyD) detectors (Leica, USA). The employed mode-locked excitation wavelength was 920 nm, for the 2P-excitation of FITC and RFP. Two GaAsP photomultiplier tube detectors were employed for detection, with band passes at 525/50 (FITC) and 595/50 (RFP). High-resolution *XYZ* stack images (1024 × 1024 pixels per *Z* step) were taken with a step size of 2.5 μm. Fluorescence intensity profiles (525/50) were acquired perpendicularly to the vessels. The decaying signal was fitted with an exponential curve [*a*·exp(±*bx*)], and the decay rate (*b*, μm^−1^) was recorded.

### Histopathology

Mice were euthanized by CO_2_ inhalation followed by cervical dislocation, and the brain was extracted and fixed in 10% formalin, paraffin-embedded, and sectioned at 5 μm. Slides were deparaffinized with xylene and rehydrated. Unstained slides were employed for the fluorescence intensity estimation of FITC within the tumor area. Stained slides with the CD31 marker were employed for blood vessel identification. The sections were counterstained with Giemsa staining. For immunohistochemistry with MMP14 staining, paraffin-embedded healthy and tumor tissue sections from the extracted mouse brains were deparaffinized, rehydrated, and then incubated with a recombinant anti-MMP14 antibody (Abcam, Cambridge, MA, USA), followed by a secondary staining using goat anti-rabbit Alexa Fluor™ 488 conjugated antibody (Thermo Fisher, Carlsbad, CA, USA). Images were acquired with an optical fluorescence microscope (Keyence BZ-X700 Series, Japan) and the green (FITC filter) fluorescence intensity in the tumor was analyzed with ImageJ.

### Statistical analyses

*T*_2_ relaxation time distributions (violin plots) of the tumors were calculated as a quantitative measure of tumor contrast enhancement. The Wilcoxon rank-sum test was performed to verify statistically significant difference between the two groups (*P* < 0.05). For the correlation study, the average value of the decay rates (*n* = 10 per mouse) were compared between the two groups (Ferumoxytol-FITC *vs.* Ferumoxytol-FITC-VDA) and correlated with the tumor *T*_2_ relaxation times estimated through contrast MRI, followed by a linear regression fit. The Pearson correlation coefficient was estimated to verify the linear dependence of the fluorescence intensity as a function of the iron concentration and the positive correlation between relaxation time and decay rate. Optical fluorescence analysis of tissues was evaluated with the Student's *t*-test.

### Dual-mode imaging approach

**Fig. 7:**
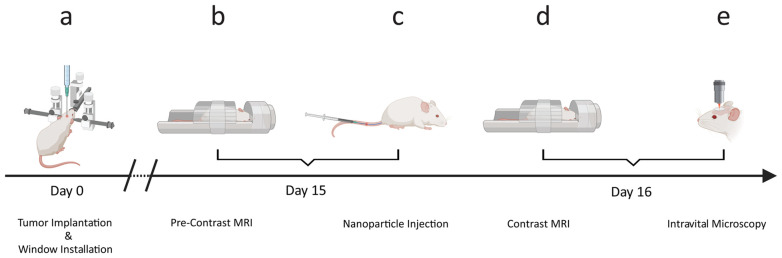
Study concept. (a) Stereotactic implantation of C6 glioblastoma cells into the right brain hemisphere and surgical implantation of a cranial glass window above the tumor implantation site. (b) Baseline pre-contrast imaging with magnetic resonance imaging (MRI). (c) Nanoparticle administration *via* intravenous injection. (d) *In vivo* post-contrast MRI. (e) Post-contrast two-photon intravital microscopy.

To enable tumor cell localization with IVM, red fluorescent protein (RFP) expressing C6 tumor cells were xenografted into the right parietal lobes of female NOD SCID gamma (NSG) mice (day 0, Fig. 7a), and an MRI-compatible glass window (5 mm diameter) was installed for intravital imaging. The xenografted tumors were allowed to develop for 15 days, followed by a baseline MRI (Fig. 7b). Directly after the baseline MRI, mice were randomized into two groups, which received a single intravenous injection of either the VDA-functionalized NPs (Ferumoxytol-FITC-VDA, 50 mgFe per kg) or Ferumoxytol-FITC (50 mgFe per kg, Fig. 7c). 24 hours later, *in vivo* dual mode imaging was achieved by scanning mice with both MRI (Fig. 7d) and IVM (Fig. 7e), exploiting the superparamagnetic properties of the iron oxide core (*T*_2_ contrast enhancer) and the conjugated fluorophore (fluorescein-5-isothiocyanate, FITC), respectively. The employed fluorophores, FITC and RFP, were chosen to match the two photomultiplier tube detectors with band passes at 525/50 nm and 595/50 nm, respectively. The combination of the two imaging techniques provided macroscopic (whole tumor) and microscopic (cellular) overview of NP tumor delivery (multiscale imaging). After the imaging sessions, the animals were euthanized, and the tumor tissue was excised for corroborative histological analysis.

## Author contributions

Conceptualization, methodology: G.M.S., D.B.M., K.N, H.E.D.L. Formal analysis, data curation, software: G.M.S., D.B.M., K.N. Writing—original draft, and visualization: G. M. S. Investigation: G.M.S., D.B.M., K.N., J.W., G.A., Z.S.V., Z.K., G.R.M., Y.S. Funding acquisition: G.M.S., H.E.D.L. Supervision, project administration, and resources: G.T., R.F., H.E.D.L. Writing—review and editing: G.M.S., D.B.M., K.N., J.W., G.A., Z.S.V., Z.K., G.R.M., E.C., L.J.P., G.T., R.F., H.E.D.L.

## Data availability

The data supporting this article have been included as part of the ESI.†

## Conflicts of interest

There are no conflicts to declare.

## Supplementary Material

NR-017-D5NR00447K-s001
