# Supplementary material for: Multimodal imaging approach to track theranostic nanoparticle accumulation in glioblastoma with magnetic resonance imaging and intravital microscopy
Source: Nanoscale. 2025 Mar 19;17(16):9986–95. doi: 10.1039/d5nr00447k (PMC11937943; doi:10.1039/d5nr00447k)
Supplement: NR-017-D5NR00447K-s001 [file NR-017-D5NR00447K-s001.pdf]

Supplementary Information (SI) for Nanoscale.
This journal is © The Royal Society of Chemistry 2025

Supplementary Information

Multimodal Imaging Approach to Track Theranostic Nanoparticle Accumulation in Glioblastoma with Magnetic Resonance Imaging and Intravital Microscopy

Giovanni M. Saladino,*[a] Dilyana B. Mangarova,[a] Kerem Nernekli,[a] Jie Wang,[a] Giacomo Annio,[a] Zahra Shokri Varniab,[a] Zubeda Khatoon,[b] Goreti Ribeiro Morais,[b] Yifeng Shi,[c] Edwin Chang,[a] Laura J. Pisani,[a] Grigory Tikhomirov,[c] Robert A. Falconer,[b] Heike E. Daldrup-Link*[a]

[a]Department of Radiology, School of Medicine, Stanford University, Stanford, CA 94305, USA.
[b]Institute of Cancer Therapeutics, Faculty of Life Sciences, University of Bradford, Bradford BD7 1DP, United Kingdom.
[c]Department of Electrical Engineering and Computer Sciences, University of California, Berkeley 94720, USA

*Corresponding authors. Email: gmsaladino@stanford.se, heiked@stanford.edu

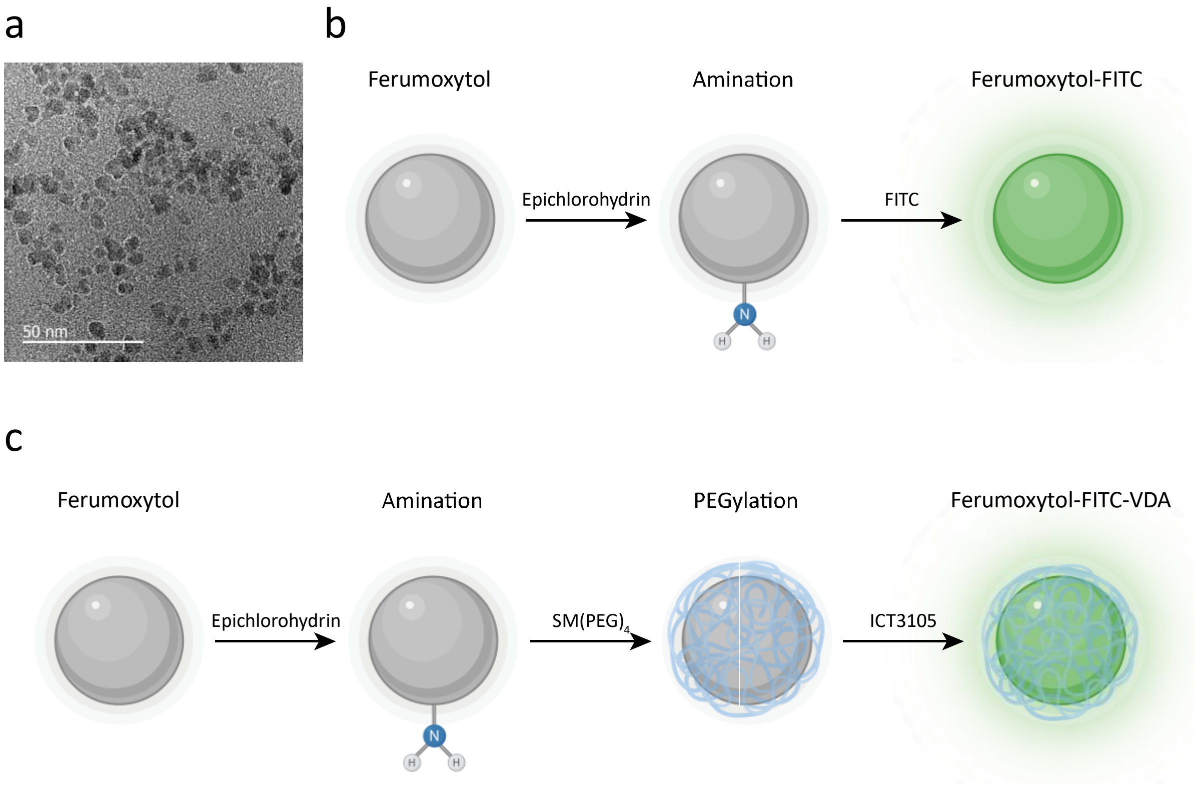

Fig. S1.

Nanoparticle Design. (**a**) Transmission electron microscopy (TEM) image of commercial Ferumoxytol, Feraheme™, used for the design of diagnostic and therapeutic nanoparticles. Scale bar, 50 nm. (**b**) Conjugation scheme for fluorescent nanoparticles: Ferumoxytol-FITC, were synthesized through amination of Ferumoxytol with epichlorohydrin and incubation with FITC. (**b**) Conjugation scheme for theranostic nanoparticles: Ferumoxytol was first aminated with epichlorohydrin and, subsequently, PEGylated with SM(PEG)$_4$, yielding maleimide-functionalized nanoparticles, which were incubated with the fluorescent therapeutic probe ICT3105, leading to Ferumoxytol-FITC-VDA nanoparticles.

Both Ferumoxytol-FITC and Ferumoxytol-FITC-VDA nanoparticles exhibited broader absorption bands between 2800 and 3600 cm^{-1}, compared to unconjugated Ferumoxytol nanoparticles. These bands were attributed to the OH and CH stretching vibrations introduced by FITC and ICT3105 on the NP surface, respectively.

The FT-IR spectra of Ferumoxytol, Ferumoxytol-FITC, and Ferumoxytol-FITC-VDA exhibited symmetric and asymmetric COO^- stretching vibrations of the polyglucose-sorbitol-carboxymethyl ether shell in Ferumoxytol, which were detected between 1550 and 1700 cm^{-1}.[28-29] Additionally, the absence of the -N=C=S stretching vibration (≈ 2010 cm^{-1}) in the spectrum of Ferumoxytol-FITC and ICT3105 indicated that FITC was completely chemisorbed, with no detectable physisorbed or free FITC present.[30-31]

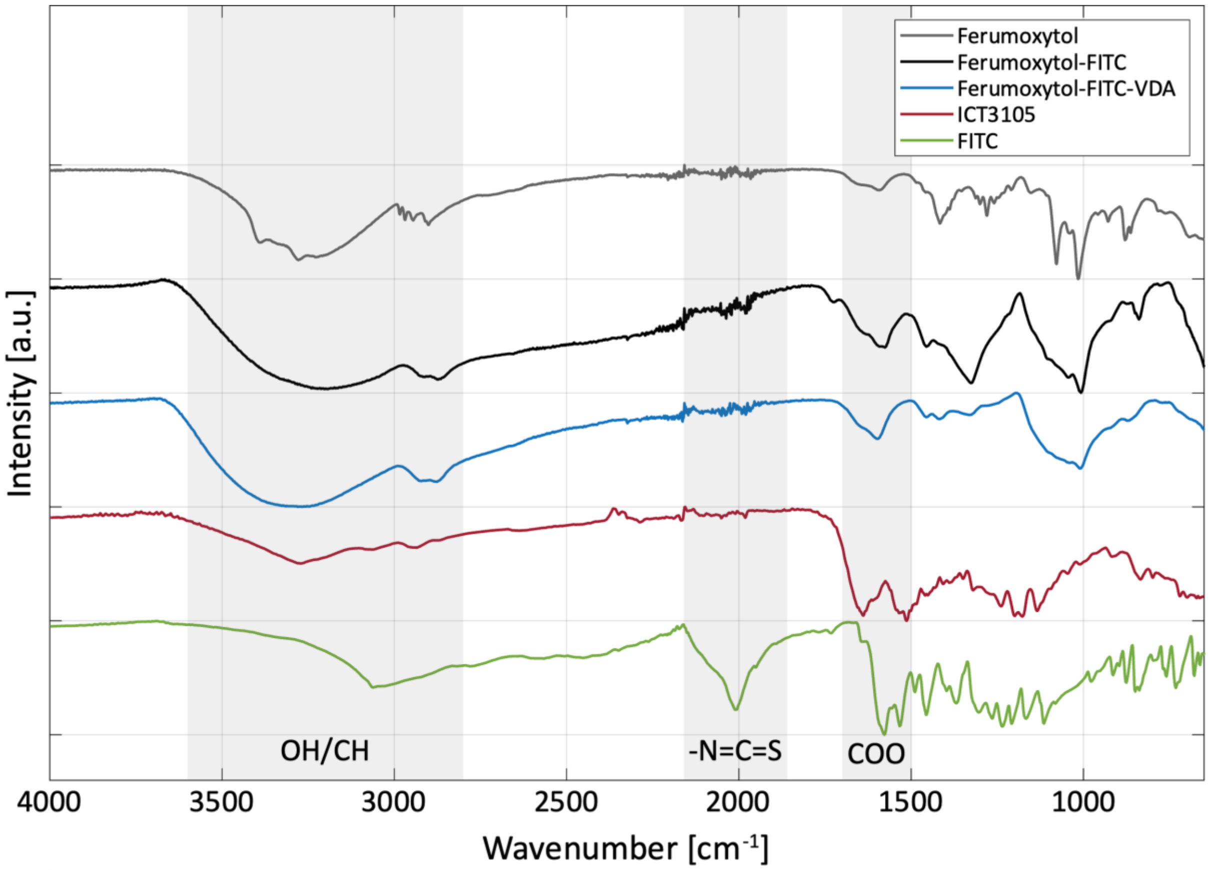

Fig. S2.

Nanoparticle Structural Characterization. FT-IR spectra of Ferumoxytol (grey), Ferumoxytol-FITC (black), Ferumoxytol-ICT (blue), ICT3105 (red), and FITC (green). The bands associated with OH/CH, NCS, and COO stretching vibrations were indicated with light-grey areas.

After 24 h exposure, the bare Ferumoxytol did not lead to any significant cytotoxicity at the tested concentrations (**Fig. S3a**), ranging from 10 ug/mL up to 1 mg/mL, consistently with the limited therapeutic effects of bare Ferumoxytol.[32-33] The fluorophore-functionalized Ferumoxytol-FITC followed a similar trend to bare Ferumoxytol, demonstrating the absence of cytotoxic effects resulting from the conjugation steps and eventual surface functionalization with FITC (**Fig. S3b**). Thus, Ferumoxytol-FITC constituted the optimal control NPs for assessing differential effects of VDA-functionalized NPs with IVM. On the other hand, the exposure of Ferumoxytol-FITC-VDA to tumor cells led to a concentration-dependent cytotoxic behavior (**Fig. S3c**), with a half maximal inhibitory concentration (IC50) of about 1 mg/mL at 24 h exposure. This therapeutic effect was ascribed to the antitumoral properties of the azademethylcolchicine-peptide conjugate (ICT3105),[17] thus confirming the potential theranostic role of Ferumoxytol-FITC-VDA.

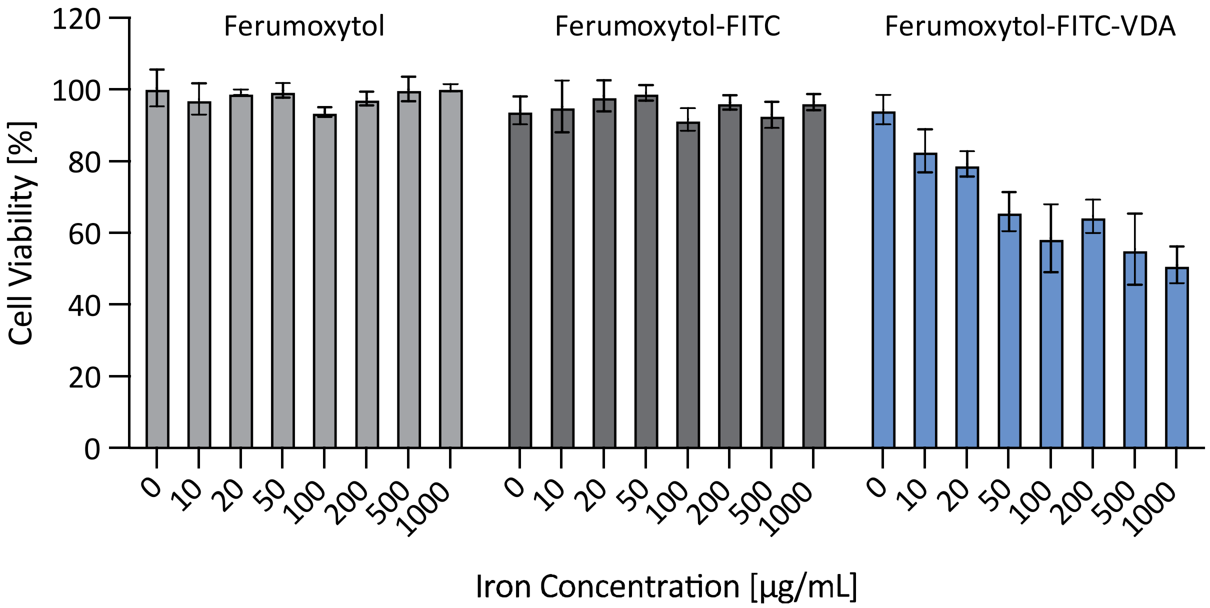

Fig. S3.
Cell studies. Cell-counting kit (CCK-8) assay on a glioblastoma cell line (C6), after exposure for 24 h to Ferumoxytol (in grey), Ferumoxytol-FITC (in black), and Ferumoxytol-ICT (in blue) with an iron concentration up to 1000 µg/mL. The cell viability values were normalized to unexposed (negative) control cells (100%). Measurements were made in triplicates (± SD).

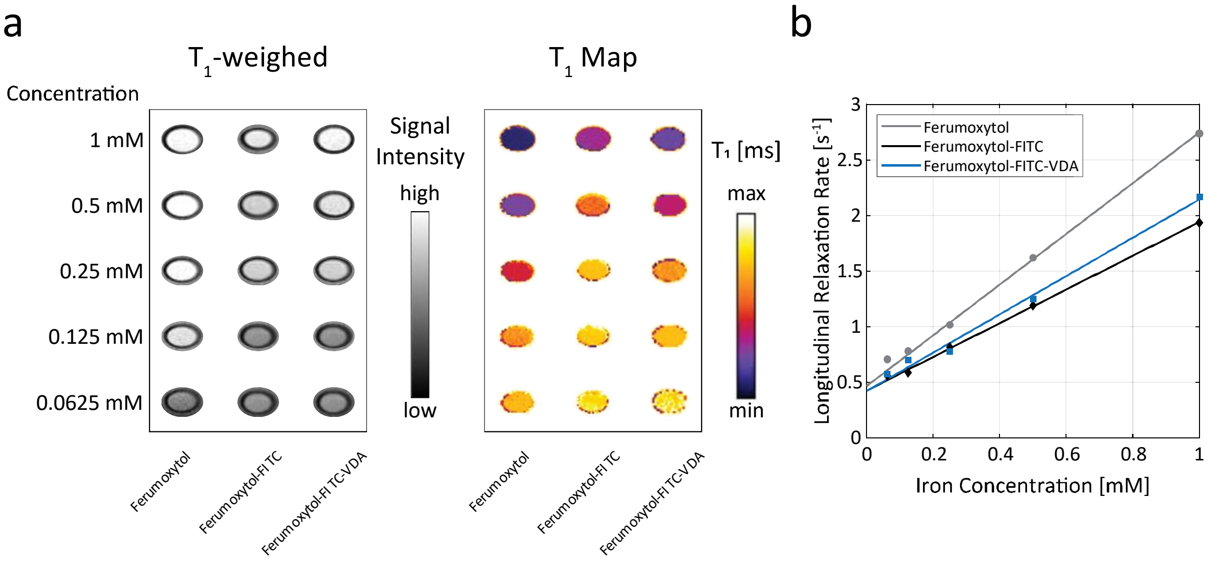

Fig. S4.

Longitudinal Relaxivity Analysis. (**a**) Representative phantom slices for T_1-weighed scans and corresponding T_1 color map showing a limited iron concentration dependency. (**b**) Longitudinal relaxation rate (R_1) as a function of iron concentration for Ferumoxytol (in grey), Ferumoxytol-FITC (in black), and Ferumoxytol-FITC-VDA (in blue).

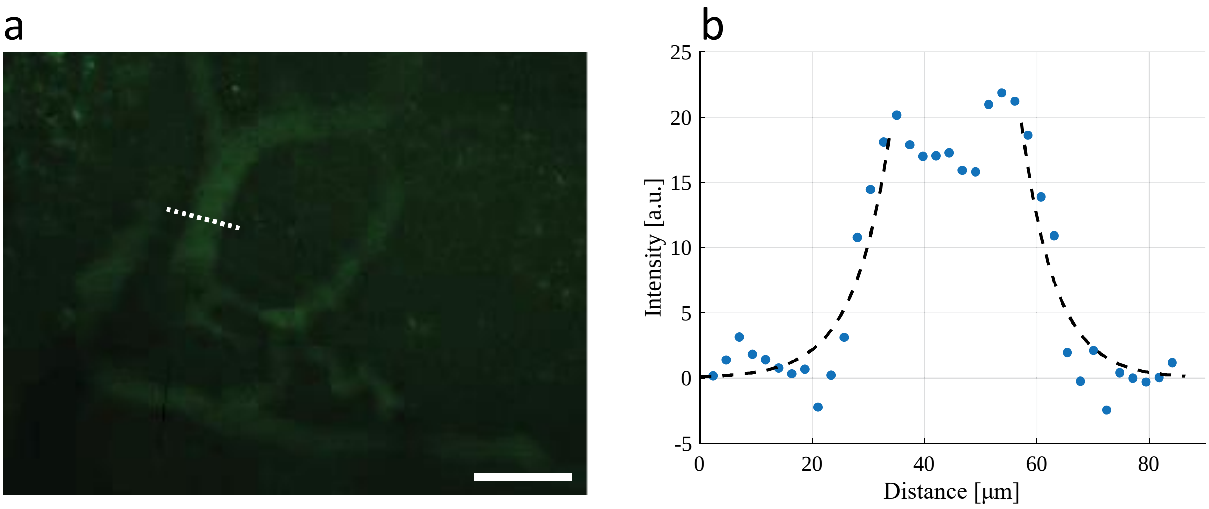

Fig. S5.

Decay Rate Estimation. (**a**) Representative two-photon intravital microscopy (IVM) image of mice administered with FITC-functionalized nanoparticles. Scale bar, 100 μm. (**b**) Intensity profile scatter points (in blue) obtained across a blood vessel (dashed white line in **a**) and subsequent exponential fitting, a·exp(±bx), of the tails (dashed black line). The estimated decay rate values, b, were recorded. $R^2 \geq 0.85$.

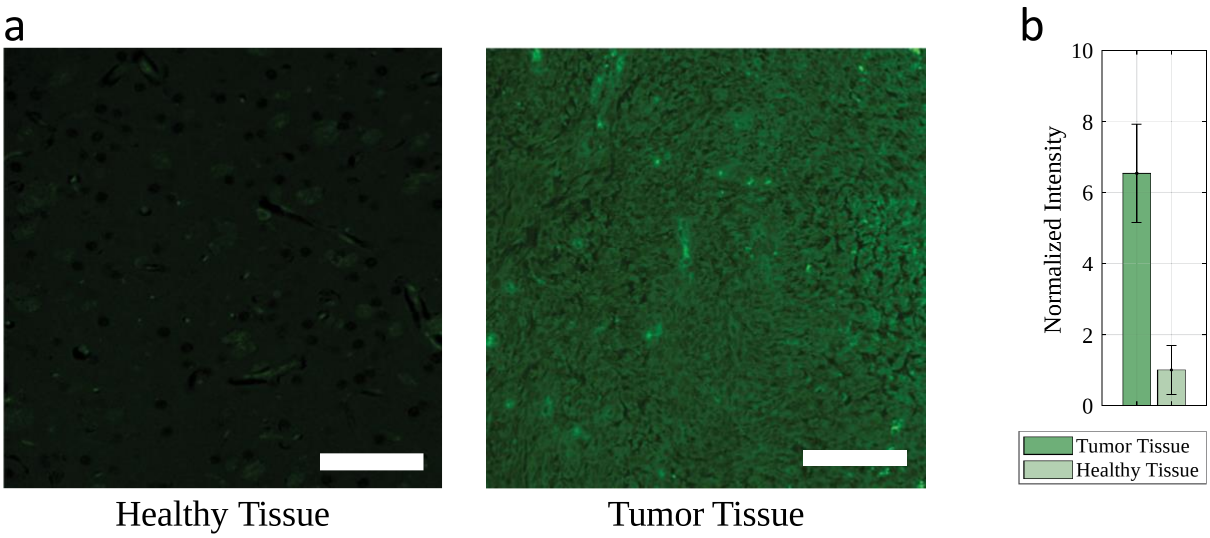

Fig. S6.

MMP14 Expression. (**a**) Immunohistochemical staining (in green) of MMP14 in slices of healthy and tumor tissues of extracted brain. Scale bars, 100 µm. (**b**) Relative quantification of the fluorescence intensity of the MMP14 expression in tumor tissue (dark green) compared to healthy (light green) tissue (normalized, ± SD).

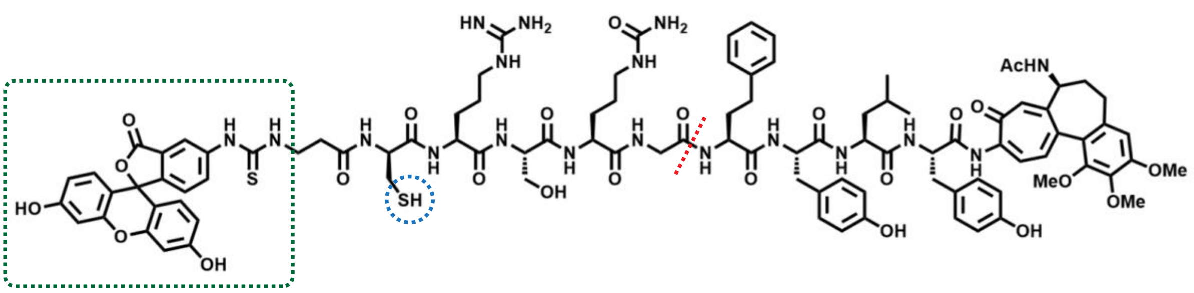

Fig. S7.

Molecular Structure of ICT3105. Matrix metalloproteinase- (MMP-)14 cleavable (specific scissile bond, line in red) vascular disrupting agent with optical fluorescence properties enabled by fluorescein isothiocyanate (FITC, square in green). Nanoparticle conjugation performed through the mercapto group (circle in blue).
